# Single-Cell Sequencing Reveals the Role of Radiation-Induced Stemness-Responsive Cancer Cells in the Development of Radioresistance

**DOI:** 10.3390/ijms26041433

**Published:** 2025-02-08

**Authors:** Zheng Shi, Cuilan Hu, Jiadi Liu, Wei Cheng, Xiaohua Chen, Xiongxiong Liu, Yanyu Bao, Haidong Tian, Boyi Yu, Feifei Gao, Fei Ye, Xiaodong Jin, Chao Sun, Qiang Li

**Affiliations:** 1 Institute of Modern Physics, Chinese Academy of Sciences, Lanzhou 730000, China; sz0326@impcas.ac.cn (Z.S.); hucuilan@impcas.ac.cn (C.H.); liujiadi@impcas.ac.cn (J.L.); chengwei@impcas.ac.cn (W.C.); chenxiaohua@impcas.ac.cn (X.C.); lxx002@impcas.ac.cn (X.L.); baoyanyu24@mails.ucas.ac.cn (Y.B.); 18093727362@163.com (H.T.); yuboyi@impcas.ac.cn (B.Y.); gaofeifei@impcas.ac.cn (F.G.); yefei@impcas.ac.cn (F.Y.); jinxd@impcas.ac.cn (X.J.); 2 Key Laboratory of Heavy Ion Radiation Biology and Medicine of Chinese Academy of Sciences, Lanzhou 730000, China; 3 Gansu Provincial Key Laboratory of Ion Beam Medicine Research, Lanzhou 730000, China; 4 College of Biopharmaceutical and Engineering, Lanzhou Jiaotong University, Lanzhou 730000, China; 5 University of Chinese Academy of Sciences, Beijing 101408, China; 6 School of Life Science and Engineering, Lanzhou University of Technology, Lanzhou 730050, China

**Keywords:** radioresistance, cancer stemness, single-cell sequencing, EGFR, hippo signaling pathway

## Abstract

Increased stemness of cancer cells exacerbates radioresistance, thereby greatly limiting the efficacy of radiotherapy. In order to study the changes in cancer cell stemness during radiotherapy, we established a radioresistance model of human non-small cell lung cancer A549 cells and obtained A549 radioresistant cells (A549-RR). We sampled the cells at different time points during the modeling process and investigated the heterogeneity of each group of cells using single-cell sequencing. Cells in the early stages of fractionated irradiation were found to be significantly up-regulated in stemness, and a subpopulation of cells producing this response was screened and referred to as “radiation-induced stemness-responsive cancer cells”. They were undergoing stemness response, energy metabolism reprogramming, and progressively differentiating into cells with more diverse and malignant phenotypes in order to attenuate the killing effect of radiation. Furthermore, we demonstrated that such responses might be driven by the activation of the EGFR-Hippo signaling pathway axis, which also plays a crucial role in the development of radioresistance. Our study reveals the dynamic evolution of cell subpopulation in cancer cells during fractionated radiotherapy; the early stage of irradiation can determine the destiny of the radiation-induced stemness-responsive cancer cells. The activation of stemness-like phenotypes during the development of radioresistance is not the result of dose accumulation but occurs during the early stage of radiotherapy with relatively low-dose irradiation. The degree of the radiation-induced stemness response of cancer cells mediated by the EGFR-Hippo signaling pathway might be a potential predictor of the efficacy of radiotherapy and the development of radioresistance.

## 1. Introduction

Non-small cell lung cancer (NSCLC) is a heterogeneous type of tumor that accounts for approximately 85% of all lung cancer diagnoses [1]. Radiotherapy, the first-line treatment for lung cancer, is greatly limited in its efficacy due to the existence of radioresistance in cancer cells [2]. Many studies have demonstrated that radioresistance is associated with tumor heterogeneity, and a very important reason for this is cancer stem cells (CSCs) [3,4,5]. CSCs are a rare subpopulation of tumor cells that are capable of self-renewal and differentiation [6,7]. Previous studies have shown that CSCs are seed cells that cause tumors to metastasize and recur, become resistant to radiotherapy or chemotherapy, and may remain latent for many years [8,9]. CSCs are characterized by metabolic reprogramming, low reactive oxygen species (ROS) production, high antioxidant capacity, and glutathione (GSH) accumulation to develop radioresistance [10]. CD133 and CD44 are the markers of CSCs, and both have been proven to correlate with radioresistance and radiotherapy prognosis [11,12,13]. In addition to inherent CSCs within the tumor, there are also radiation-induced CSCs, i.e., cells whose stemness properties are enhanced by irradiation. The formation and enhancement of the CSC phenotype is mainly associated with epithelial–mesenchymal transition (EMT), which enhances the invasion and metastasis of cancer cells and weakens the tumor-killing effect of radiotherapy [14,15]. In NSCLC cells, it has been demonstrated that radioresistance is associated with an enhanced CSC phenotype. For example, it has been proved that radioresistance in NSCLC is correlated with enhanced EMT, the CSC phenotype, and the activation of the Prx-1/NF-kB/iNOS signaling pathway [16]. Attenuated LKB1-SIK1 signaling promotes EMT and radioresistance in NSCLC cells [17]. LINC01224/ZNF91 drives radioresistance regulation by promoting stem cell-like properties in NSCLC cells [18]. SOX2 is involved in the tumorigenesis, progression, and maintenance of cell stemness and regulates radioresistance in NSCLC by promoting cell de-differentiation [19].

Epidermal growth factor receptor (EGFR), a tyrosine kinase-type receptor of the ErbB family, is overexpressed in 40–80% of NSCLC patients and is associated with poor prognosis [20,21]. There are many studies demonstrating that EGFR can support the generation and maintenance of CSC-like cells. SALL4 [22] and CD166 [23] can be activated by the EGFR/ERK1/2 signaling pathway to regulate CSC formation. There are several mutated forms of EGFR, the most prominent of which is EGFRvIII [24]. The aberrant expression of EGFRvIII regulates cancer progression [25] and modulates STAT3 signaling [26,27], which is involved in the control of cell stemness [28]. EGFRvIII can increase SOX2 expression, thereby promoting the self-renewal of prostate CSCs [29]. Several studies have also evidenced the regulatory role of the EGFR-induced CSC phenotype for radioresistance. Radioresistance in KRAS mutant lung cancer is due to stem-like properties mediated by the Osteopontin-EGFR pathway [30]. The EGFR-PKM2 axis can modulate stem cell-like phenotypes in radioresistant cancer cells [31].

The Hippo pathway plays an important role in organ development and regeneration [32]. Through inhibiting Yes-Associated Protein (YAP) and transcriptional coactivator with PDZ-binding motif (TAZ), the Hippo pathway regulates cell proliferation, apoptosis, and stemness in response to a variety of extracellular and intracellular signals [33]. Firstly, the regulatory effects of the Hippo pathway on stem cells are widely known. For instance, TAZ is able to confer stem cell-associated properties to cancer cells [34] and can promote EMT [35]. The RNA-binding protein complex LIN28/MSI2 enhances cancer stem cell-like properties by regulating Hippo-YAP1 signaling [35]. FZD10 [36] and PFKFB3 [37] can regulate stem cell properties and the Hippo signaling pathway. Secondly, it has been shown that the Hippo pathway enhances radioresistance in cancer cells. Esophageal cancer cells with a strong expression of TAZ survive longer after radiation than cells with reduced TAZ expression [38]. CD155 enhances radioresistance by regulating the Hippo-YAP pathway [39]. The interaction of CD146 with integrin β1 activates LATS1-YAP signaling, promotes the emergence of cell stemness-like features in breast cancer cells, and induces radioresistance [40].

Radiotherapy is poorly effective because CSCs exacerbate the development of radioresistance. Many patients experience tumor recurrence and develop radioresistance after radiotherapy. Methods or molecular markers that can predict the effect of radiotherapy at an early stage can guide medical doctors to make timely adjustments and modifications to a patient’s therapeutic plan, avoiding ineffective treatment of the patient and the waste of medical resources. Currently, there are no mature protocols and molecular indicators that have an early prognostic effect applied in the clinic. The reason for this may be the lack of dynamic follow-up of the process of radioresistance development and the lack of corresponding studies on the role and function of the stemness response in this dynamic process. Therefore, we simulated the course of radiotherapy in NSCLC from early to late stages until recurrence (developing stable radioresistance) using the NSCLC cell line A549 and analyzed samples at time points in the process using single-cell sequencing technology. The aim of our study was to find a cluster of cells with a stem-like response to radiation during heterogeneous changes and investigate their differentiation trajectories and drivers to seek molecular indicators that can predict the effects of radiotherapy early.

## 2. Results

### 2.1. Modeling and Validation of Radioresistance in A549-RR Cells

#### 2.1.1. In Vitro Experimental Procedure and Validation

**Figure 1:**
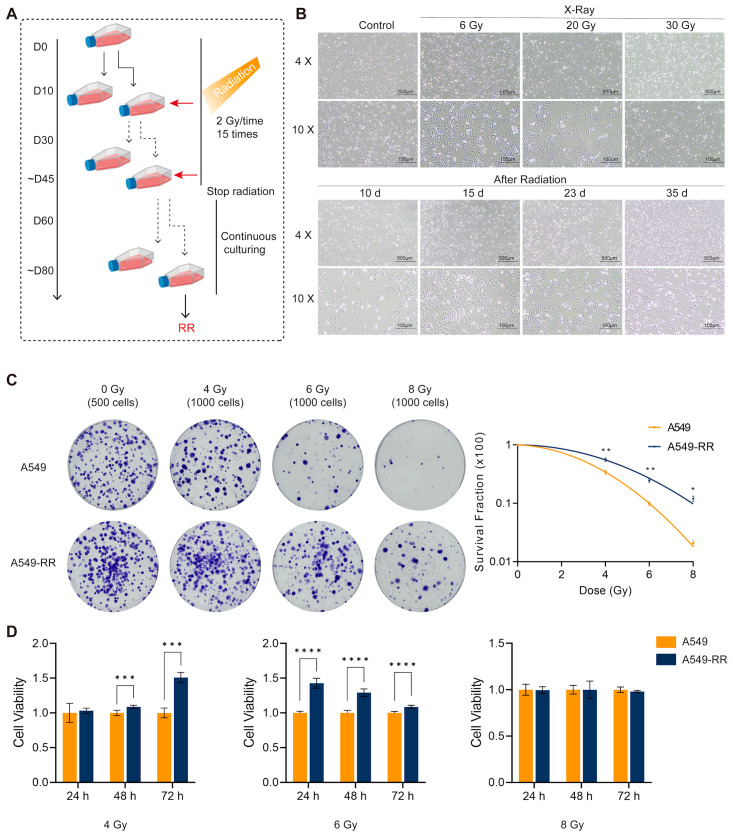
Radioresistance(RR) modeling and in vitro validation. (**A**) The radioresistance modeling process. (**B**) Cell morphology recordings during the model building. (**C**) Results of the colony formation experiments after 0, 4, 6, and 8 Gy irradiation. (* *p* < 0.1; ** *p* < 0.01). (**D**) CCK-8 results after 0, 4, 6, and 8 Gy irradiation. (*** *p* < 0.001; **** *p* < 0.0001).

**Figure 2:**
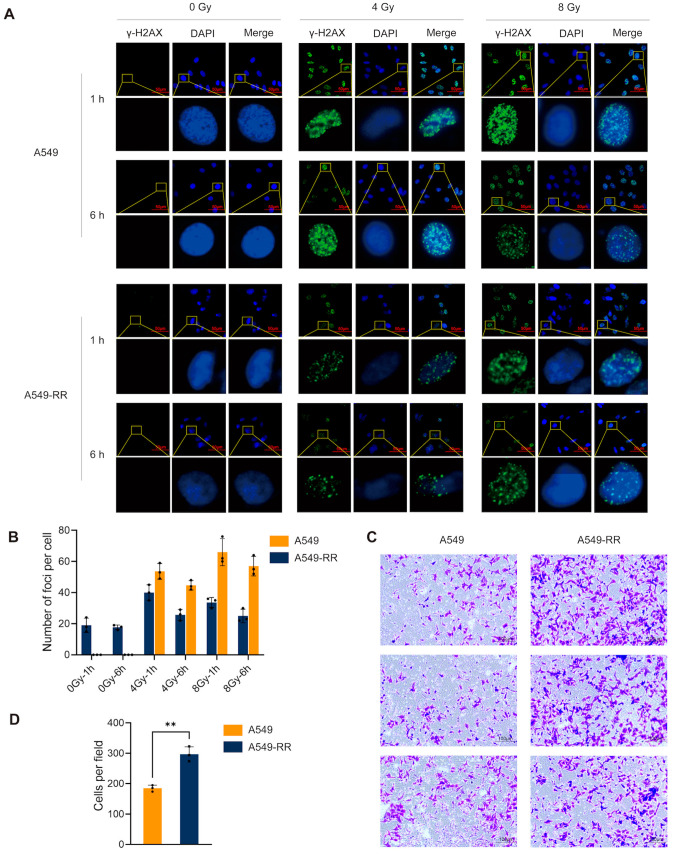
In vitro validation of radioresistant cells. (**A**,**B**) Immunofluorescence detection of the degree of DNA damage after irradiation and the result of quantification. The green dots represent γ-H2AX foci, i.e., DNA break sites. The black dots in the column chart represent the distribution of the data. (**C**,**D**) Images and quantitative results of the Transwell experiment. Cells that were stained in violet are cells that cross the stromal gel. The black dots in the column chart represent the distribution of the data. (** *p* < 0.01).

The radioresistance modeling process is shown in Figure 1A. X-ray irradiation mimicked a clinical treatment course with 2 Gy/dose, for a total dose of 30 Gy. During the treatment course, cells were cultured each time until they reached 80% confluence or more. After the accumulated dose reached 30 Gy, the irradiation was stopped, and the cells were cultured for more than 30 days to obtain radioresistant cells (A549-RR). We can find that with the increase in cumulative irradiation dose, the cell morphology changed significantly, showing distorted and overspreading patterns. After continuous culture following cumulative irradiation of 30 Gy, it could be observed that the cell morphology recovered to nearly its initial state after one month. The radioresistance of A549-RR cells was verified after the model establishment was completed. The results of the colony formation assay are shown in Figure 1C. The 0 Gy group was inoculated with 500 cells/dish, while all irradiated groups were inoculated with 1000 cells/dish. The graph on the right shows the survival curves of the cells. After 4, 6, and 8 Gy treatments, the A549-RR group had more clone sites than the A549 group. The CCK-8 results are shown in Figure 1D. Clearly, the cell viability of A549-RR cells was better than that of A549 cells after irradiation with 4 Gy and 6 Gy. As shown in Figure 2A,B, the immunofluorescence experiments demonstrated that the cells in the A549-RR group had significantly less DNA damage than those in the A549 group after irradiation with 4 Gy and 8 Gy, and the damage of A549-RR cells was reduced more at 6 h. Collectively, all of the above results indicated that A549-RR cells had significant radioresistance and improved DNA damage repair ability. The Transwell assay results are shown in Figure 2C,D. The number of cells crossing the stromal gel in the A549-RR group was significantly higher compared to the A549 group after filling the cells into the chambers for 24 h, indicating that A549-RR cells had stronger invasive ability and a malignant phenotype.

#### 2.1.2. In Vivo Validation

**Figure 3:**
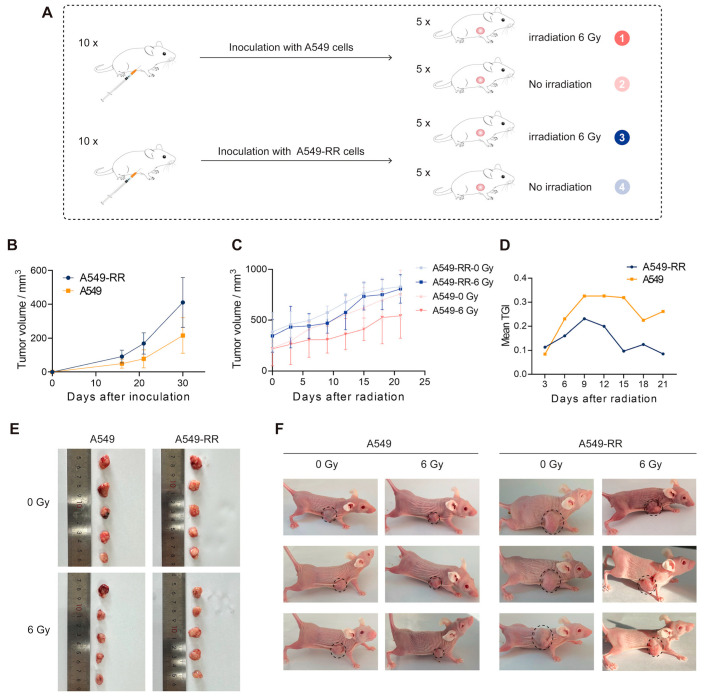

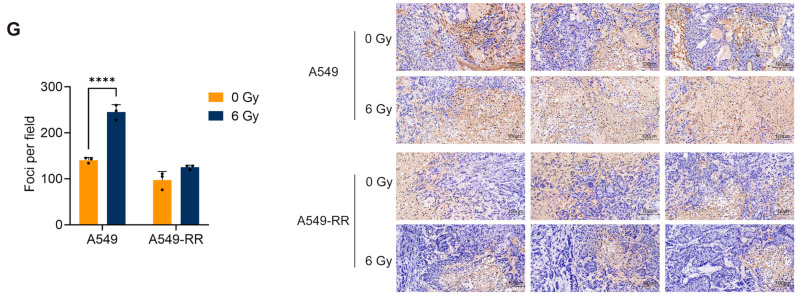
In vivo validation of radioresistance. (**A**) Presentation of the grouping of nude mice with different treatments. (**B**) Tumor volume after inoculation. (**C**) Tumor volume after irradiation. (**D**) Mean TGI in the A549-RR and A549 groups. (**E**) Tumor size in the different groups after dissection. (**F**) Tumor size in subcutaneously loaded mice before dissection. Tumors in mice are marked with dash circles. (**G**) Tunel staining and quantitative results of tumor sections in each group after dissection. The brown dots represent apoptotic nuclei. The black dots in the column chart represent the distribution of the data. (**** *p* < 0.0001).

To verify the radioresistance of A549-RR cells in vivo, we inoculated A549 and A549-RR cells under the skin of nude mice and divided them into irradiated and non-irradiated groups, as shown in Figure 3A, for a total of four groups with five mice each. The results showed that the subcutaneous tumor growth rate of the A549-RR group was significantly faster than that of the A549 group, as shown in Figure 3B. Figure 3C–F illustrate that X-ray irradiation did not have a noticeable effect on the volume of tumors in the A549-RR group but showed a markedly inhibitory effect on the A549 group, and the mean tumor growth inhibition (TGI) value also showed an obvious difference between the two groups. As shown in Figure 3G, tunel staining of the tumor tissues demonstrated that the A549 group presented more apoptotic nuclei compared with the A549-RR group tumors after radiation. For the non-irradiated group, the A549 group had more apoptotic nuclei than the A549-RR group. The above experiments demonstrated that subcutaneous tumors formed by A549-RR cells were radioresistant and more malignant.

### 2.2. Single-Cell Sequencing Revealed the Role of Radiation-Induced Stemness-Responsive Cancer Cells in the Development of Radioresistance

#### 2.2.1. Cell Clustering and Cell Type Analysis

**Figure 4:**
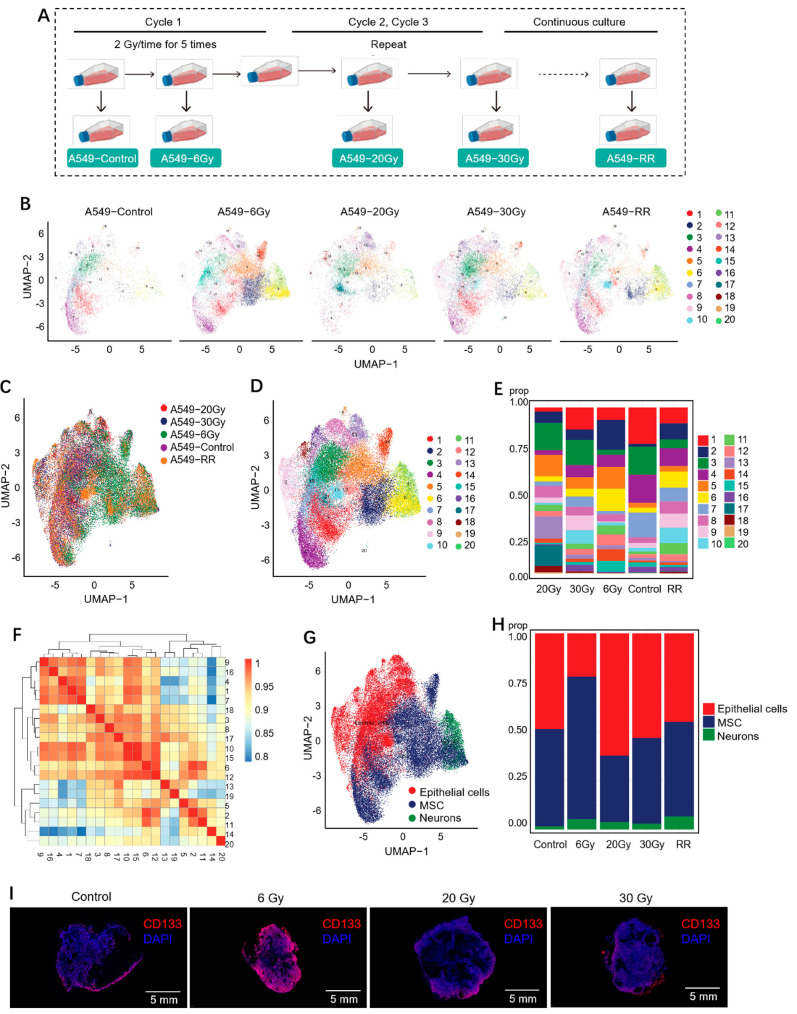
Data from single-cell sequencing. (**A**) Demonstration of the sampling process during modeling of radioresistant cells. (**B**) UMAP plot for each sample after QC. (**C**) Distribution of different samples on the UMAP plot. (**D**) Distribution of different clusters on the UMAP plot. (**E**) Proportion of clusters in different samples. (**F**) Correlation between each cluster. (**G**) Distribution of different cell types in the UMAP plot. (**H**) Proportion of different cell types in different samples. (**I**) Immunofluorescence results of CD133 in subcutaneous tumor-bearing mice irradiated with different cumulative doses.

As shown in Figure 4A, we took a total of five samples in the process of radioresistance modeling, i.e., A549-Control, A549-6Gy, A549-20Gy, A549-30Gy, and A549-RR. They corresponded to the cells when the cumulative dose reached 0, 6, 20, and 30 Gy and after the formation of stable radioresistance, respectively. To ensure the timeliness of the samples, single-cell sequencing operations were performed in a timely manner after each individual sample was taken. Finally, a total of 59,320 cells across the five samples were identified, and they were clustered into 20 clusters using the Uniform Manifold Approximation and Projection (UMAP) dimensionality reduction method, as shown in Figure 4B–D. Each point in the figure represents a cell. The cells marked with the same color in Figure 4B,D were the cells of the same clusters; the cells marked with the same color in Figure 4C originated from the same sample. Figure 4E demonstrates the percentage of different clusters in each sample. The overall correlation between cell clusters was assessed (Figure 4F), with redder colors representing more correlation. The results of the cell clustering analysis showed strong heterogeneity already in the A549-6Gy group. From the results of cell types (Figure 4G,H), mesenchymal stem cell (MSC)-like cells had the highest percentage in the A549-6Gy group. To verify the point, we irradiated mice with subcutaneous tumors with X-rays at 2 Gy/time, leading to total doses of 6 Gy, 20 Gy, and 30 Gy, and sampled them separately to detect their expression levels of the stem cell marker CD133. The results are shown in Figure 4I. The highest fluorescence intensity appeared after the cumulative metered dose of 6 Gy irradiation, which is consistent with the cell type identification result. Based on the above results, we could conclude that after cumulative 6 Gy irradiation, both cells in vitro and in vivo developed an enhanced stemness-like phenotype. However, at the level of cell clusters, we still need to explore more to find a group of cells that dominate this response.

#### 2.2.2. Exploration of Stemness-Responsive Cell Subpopulations

**Figure 5:**
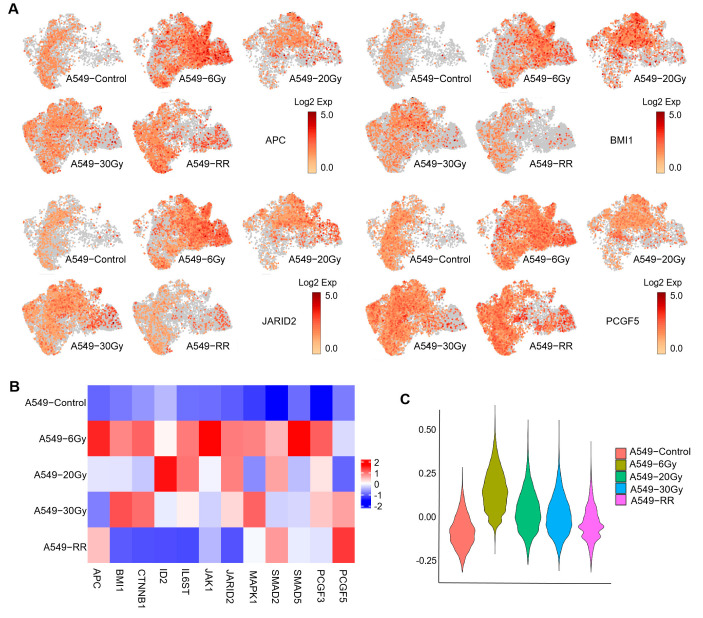

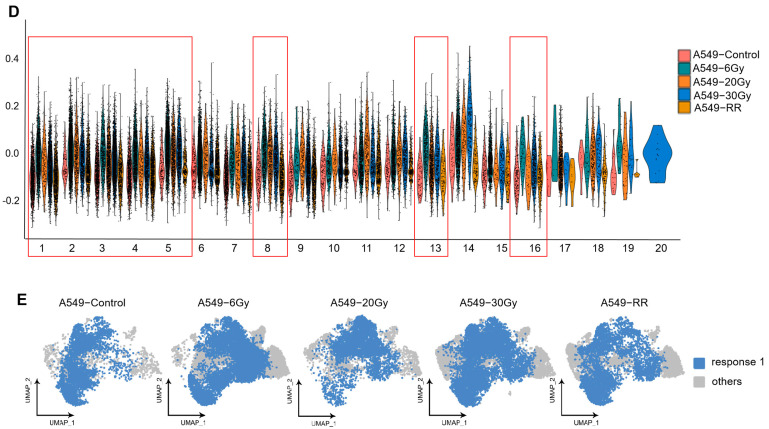
Expression of CSC-related genes by each group and cluster. (**A**) Presentation of the expression and distribution of the four stemness-related genes in the overall sample in the UMAP plot. Redder colors indicate higher expression. (**B**) Heatmap of the expression of stemness-related genes in each group. Redder colors indicate higher expression and bluer colors indicate lower expression. (**C**) Violin plots of the overall expression of stemness-related genes in each group. (**D**) Violin plots of the expression of stemness-related genes by group and corresponding clusters. The red boxes mark the clusters that correspond to the highest expression in the A549-6 Gy group. These clusters are called Response 1. (**E**) Distribution of Response 1 across groups in the UMAP plot.

Firstly, we verified the expression levels of stem cell-related genes in different samples using some signature genes under enrichment of stem cell-related pathways. We found that stem-like gene expression was significantly higher in the A549-6Gy group compared to the A549-Control group, presenting a decreasing trend with increasing doses, as shown in Figure 5A–C. However, from the expression distribution of the genes presented in Figure 5A, we could see that the stem-like genes were not enriched in a specific cluster (see Figure S1 in the Supplementary Materials for all stem cell-related genes expressed on the UMAP map). In order to find out which clusters showed the most pronounced stemness-like expression in the early stage of the radiotherapy course, i.e., when the cumulative dose reached 6 Gy, we used the expanded set of genes under the stem cell-related pathway to score each cluster, and the results are shown in Figure 5D (see Table S1 in the Supplementary Materials for the gene set for scoring). From these, we selected the clusters (clusters 1–5, 8, 13, 16) with the same trend of the highest strongest stem-like phenotype in the A549-6 Gy group and referred to them as Response 1. The distribution of Response 1 across samples is shown in Figure 5E. This suggested that many cancer cells could respond to irradiation in a stem-like manner. We referred to the cell population represented by Response 1 as “radiation-induced stemness-responsive cancer cells”.

#### 2.2.3. Pseudotime Analysis of Response 1

**Figure 6:**
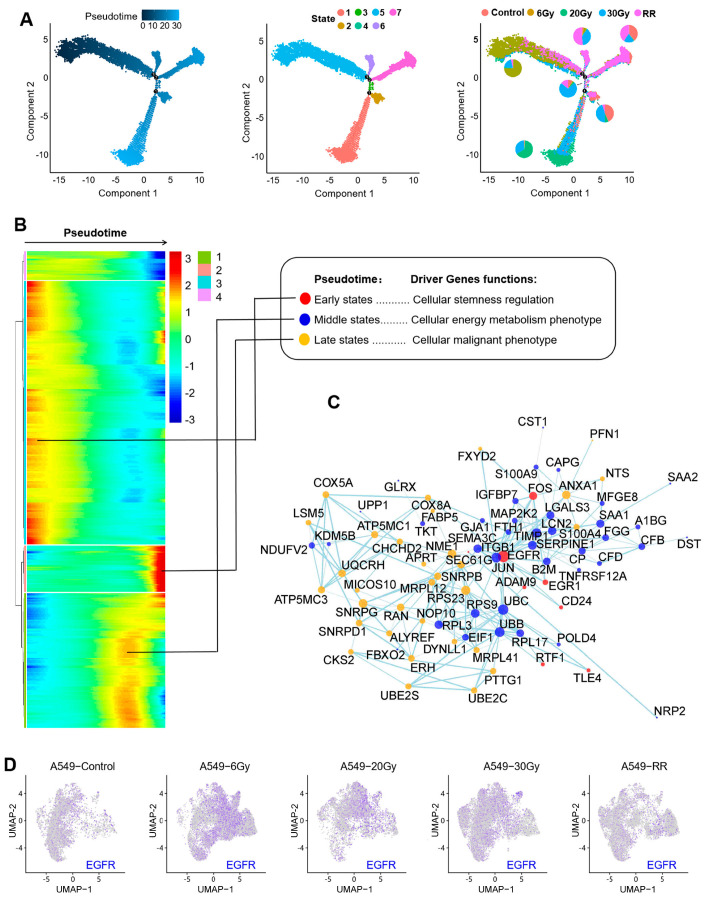
Results of the pseudotime analysis for Response 1. (**A**) The plots from left to right show the timeline of the pseudotime, the distribution of each state, and the distribution of each group, respectively. The black dots represent locations on the differentiation trajectory that begin to have different directions. (**B**) Heatmap of highly expressed driver genes on the timeline. Red color indicates higher expression while blue color indicates lower expression. (**C**) Results of gene interaction analysis. (**D**) Distribution of EGFR in each group in the UMAP plot.

To find the differentiation trajectories in Response 1, we performed a pseudotime analysis. Figure 6A represents, from left to right, the direction of pseudotime from dark blue to light blue, the differentiation process of cells on the pseudotime axis with seven different states, and the distribution of each group on the timeline, respectively. The above three figures have a corresponding relationship. We could see that the differentiation trajectory of Response 1 started from the A549-6Gy group and had a sample-dependent pattern, with most of the endpoints being radioresistant (RR) cells. This suggested that in the early stages of radiotherapy, radiation-induced stemness-responsive cancer cells have the ability or destiny to differentiate into radioresistant cells, which is important information.

To specify the genes that play a significant role in the differentiation trajectory of the pseudotime axis, as shown in Figure 6B, we selected stemness-related genes in the early stage of the timeline and genes with higher expression in the middle and late stages based on the heatmap. The highly expressed genes in the middle stage of differentiation were found to be mainly associated with the cellular energy metabolism phenotype, while the later stage was mainly associated with the malignant phenotype. As shown in Figure 6C, to clarify which of these genes play more important roles and how they interact with each other, we performed gene interaction analyses on highly expressed genes in each period. The results showed that EGFR was a core gene of the radiation-induced stemness-like response and was involved in the formation of radioresistance by affecting metabolic respiration-related genes and malignancy-related regulation. And, as shown in Figure 6D, we enriched the expression of EGFR on the UMAP plot, and the results showed that the A549-6Gy group was relatively enriched.

#### 2.2.4. Functional Enrichment of Response 1

**Figure 7:**
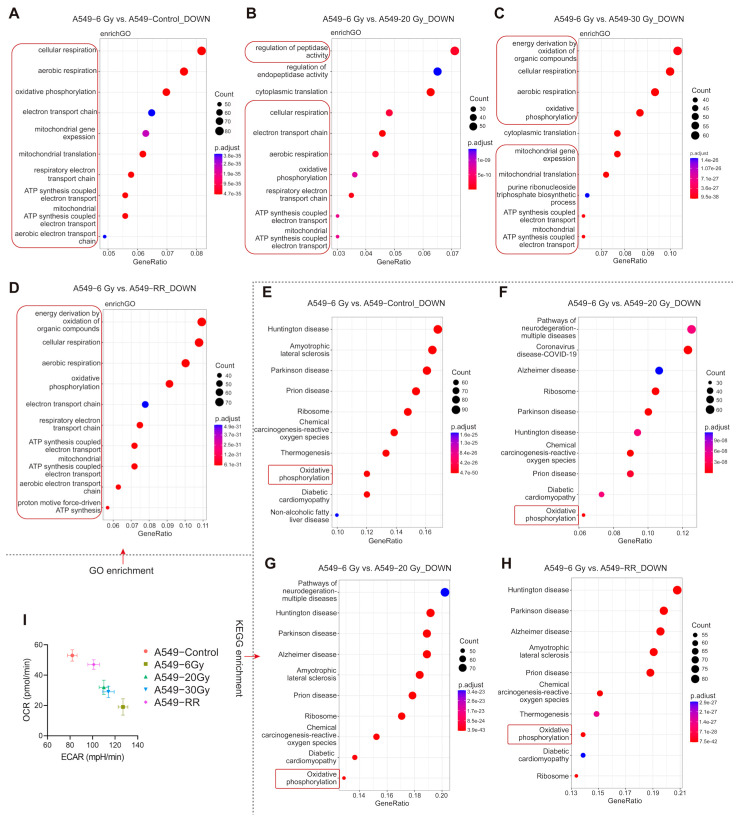
Functional enrichment between groups in Response 1. (**A**–**D**) GO enrichment analysis of the A549-6Gy group compared to the other groups with down-regulation. (**E**–**H**) KEGG enrichment analyses of the A549-6Gy group compared to the other groups with down-regulation. (**I**) Energy metabolism phenotypes of each group.

It has been shown that various types of CSCs are dependent on glycolysis for energy, which means that undergoing energy metabolic reprogramming through oxidative phosphorylation to aerobic glycolysis is a common feature of CSCs [41,42,43,44]. As shown in Figure 7A–H, we found that Response 1 was very significantly down-regulated in aerobic respiration in the A549-6Gy group compared to all other four groups. We boxed the relevant items in red, and it could be seen that almost all the top ten items in the Gene Ontology (GO) enrichment analysis were associated with aerobic respiration down-regulation and mitochondrial function; significant oxidative phosphorylation down-regulation was also seen in the Kyoto Encyclopedia of Genes and Genomes (KEGG) enrichment analysis in the corresponding groups. To verify whether the cells underwent energy metabolic reprogramming, we examined the energy metabolism phenotypes of each group. The basal oxygen consumption rate (OCR) and extracellular acidification rate (ECAR) were tested using a Seahorse analyzer. The results are shown in Figure 7I. Compared with the A549-Control group, the A549-6Gy group showed a significantly higher ECAR and lower OCR, representing a clear shift from oxidative respiration to glycolysis after irradiation at a cumulative 6 Gy dose. In contrast to the other three groups, which all had varying degrees of recovery of OCR, the A549-6Gy group had the most pronounced phenotypic shift in energy metabolism. These results further support that the cells in Response 1 showed very distinct phenotypic changes, reprogramming of energy metabolism, and acquired stem cell-like properties after a cumulative dose of 6 Gy irradiation.

**Figure 8:**
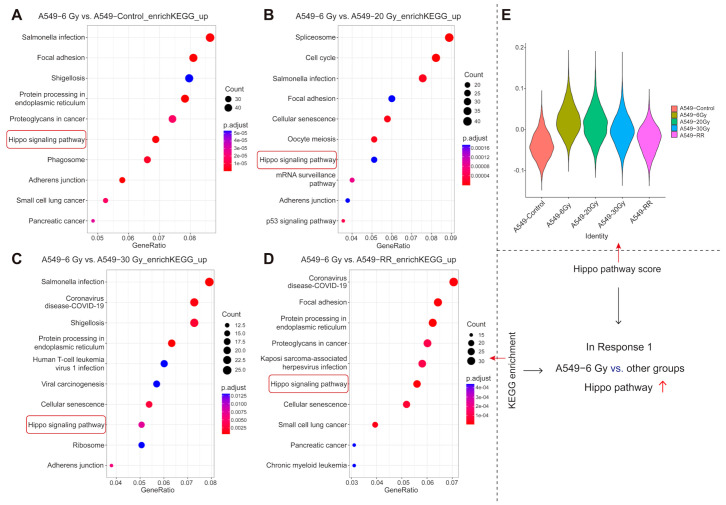
Activation of the Hippo pathway by the A549-6Gy group vs. the other groups. (**A**–**D**) KEGG enrichment analysis of the A549-6Gy group compared to the other groups with up-regulation. (**E**) Violin plots of the scoring of groups with genes related to the Hippo pathway.

As shown in Figure 8A–D, from the results of the KEGG enrichment, we found that Response 1 showed an up-regulation of the Hippo signaling pathway in the A549-6Gy group compared to the other four groups. We then scored the groups using the Hippo pathway gene set, which indicated that the A549-6Gy group showed a notable enhancement compared to the A549-Control group and exhibited a decreasing trend with the accumulation of the dose (see Table S2 in the Supplementary Materials for the set of genes used for scoring the Hippo pathway). This proved that there was an obvious up-regulation of the Hippo pathway in the cancer cells with the cumulative dose of 6 Gy irradiation.

### 2.3. The EGFR-Hippo Pathway Regulated the Radiation-Induced Stemness Response of A549 Cells Resulting in Radioresistance

**Figure 9:**
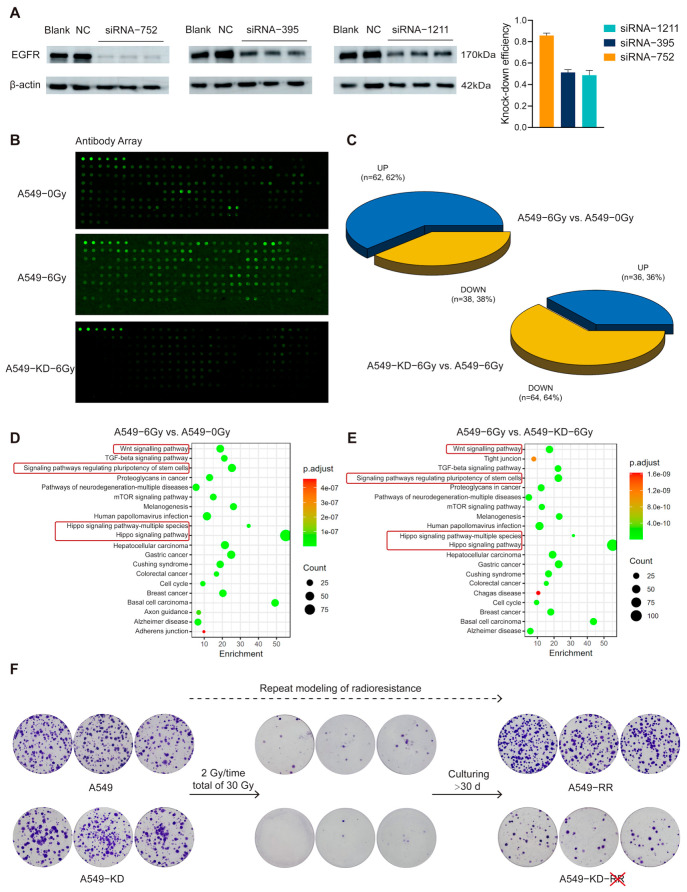
Biologically validated experimental results. (**A**) Comparison of knockdown efficiency of EGFR by three different si-RNAs. (**B**) Raw fluorescent signals from protein screening array experiment. (**C**) Pie chart comparing the number of up- and down-regulations in top 100 differential proteins of the Hippo pathway protein screening array. (**D**,**E**) KEGG enrichment analysis derived from differential proteins. (**F**) Results of the colony formation experiments during repetition of the radioresistance model for A549 and A549-KD.

To verify whether the activation of the Hippo pathway by low-dose radiation is mediated through EGFR, firstly, three different siRNAs were used for EGFR knockdown (KD), and siRNA-752 showed the most significant knockdown efficiency (Figure 9A). We started X-ray irradiation at 24 h after EGFR knockdown, 2 Gy/time for 3 consecutive days, with a cumulative measurement of 6 Gy. Cell precipitates were collected and used in the protein screening array experiments to determine the expression of Hippo pathway-related proteins in each group. Figure 9B shows the raw fluorescence signals of protein expression for each group (see Supplementary Materials Table S3 for the map of the antibodies corresponding to the antibody array). Figure 9C represents the statistical results of the number of up-regulated and down-regulated differential proteins in the top 100 compared between the two groups (see Figure S2 in the Supplementary Materials for results of the top 100 differential proteins with protein names compared between the two groups). The numbers of up-regulated and down-regulated proteins in A549-6Gy compared to A549-0Gy were 62 and 38, respectively; in A549-KD-6Gy compared to A549-6Gy, the numbers were 36 and 64, respectively. This indicated that the Hippo pathway was activated after irradiation in A549 cells; however, this activity was no longer significant when EGFR was knocked down. This result suggested that EGFR played a critical role in the activation of the Hippo pathway in A549 cells induced by low-dose radiation. Meanwhile, we performed KEGG enrichment for the differential proteins therein. As shown in Figure 9D,E, the A549-6Gy group showed a trend of an up-regulation of stem cell pluripotency-related pathways along with activation of the Hippo pathway compared with the A549-0Gy group and the A549-KD-6Gy group. This suggested that cells in the A549 group acquired significant stemness-like features after irradiation at a cumulative dose of 6 Gy; however, when EGFR was knocked down, irradiation-induced stemness-like features decreased in comparison with non-knocked down cells.

To verify whether radioresistance could still be developed after EGFR was knocked down, we started to repeat the radioresistance model 24 h after the transfection of siRNA-752 by the same method as shown in Figure 1A in the previous subsection, with 2 Gy/time, and, after accumulating a dose of 30 Gy, the cells were cultured continuously for about 30 days. The results of the colony formation experiments are shown in Figure 9F. Before the beginning of irradiation, both A549 and A549-KD could grow obvious clone sites. After the cumulative dose of 30 Gy irradiation, both groups of cells were severely damaged, and we could see that A549 clonal sites were few, while A549-KD was even fewer and nearly absent. After about 30 days of culture under normal conditions, A549 cells were able to form radioresistance (A549-RR) according to the verified model, growing clear and more numerous clonal sites; however, A549-KD was unable to grow clonal sites like A549-RR after the same culture, indicating that it did not develop radioresistance. This suggested that EGFR had a major role in the generation of radioresistance.

The above results indicated that radiation-induced stemness-responsive cancer cells regulated stemness by activating the radiation-induced EGFR-Hippo pathway and played a key role in the development of radioresistance.

## 3. Discussion

Our study successfully established a model of radioresistance in A549 cells that mimicked a clinical radiotherapy treatment and verified that A549-RR cells had significant radioresistance using in vivo and in vitro experiments. We retained samples at each cumulative dose point during the modeling process and promptly put the samples to test. Using single-cell sequencing, we discovered the dynamic evolution of cell subpopulation in cancer cells during radiotherapy and found a subpopulation of cells that produced stemness in response to radiation and that only the enhancement of stem responsiveness gave the cancer cells a tendency to develop into radioresistant cells. We refer to this subpopulation of cells as “radiation-induced stemness-responsive cancer cells” and believe they have the destiny to differentiate into radiation-resistant cells from the early stages of radiotherapy. The diversity of cells produced by the differentiation of stem cells gives the whole population of cells the ability to resist radiation. This suggests that radiation-induced stem-responsive cells are the main source of late-acquired radiation-resistant cells, in addition to the pre-existing population of innate radiation-resistant cells.

CSCs are a rare population in tumors, numbering less than 0.1% [45,46,47]. Targeting CSCs to attack tumors has long been of interest to researchers, and new targets are constantly being discovered. For example, Lgr5, a G protein-coupled receptor containing leucine-rich repeat sequences, is highly expressed in different types of cancers and is a unique marker for CSCs. It was found that the growth of Lgr5+ cells can be controlled by PTEN/AKT and Wnt/β-catenin pathways, and Lgr5 has emerged as a potential therapeutic target for the treatment of hepatocellular carcinoma [48,49]. CSCs have been shown to be involved in the development of tumor radioresistance [8,13,14]. Ionizing radiation triggers ROS production and oxidative stress in tumor cells, leading to DNA damage [50]. The cellular response to radiotherapy depends on the ability to clear ROS and repair DNA damage [51]. CSCs have an efficient ROS scavenging system and generally low ROS levels, which may explain the higher resistance to radiotherapy in the CSC population of different tumor entities [52,53,54]. In past studies, changes in CSC content can affect the development of radioresistance by interfering with DNA repair mechanisms and cell cycle redistribution [55]. Biomarkers of CSC predict radiotherapy outcomes in certain tumor entities [56]. So, it is a commonly known fact that CSCs are inherently radioresistant. However, studies have also identified a relationship between CSCs and acquired radioresistance, demonstrating that in oral squamous carcinoma, radioresistance is induced by the up-regulation of a stem cell-like phenotype in cancer cells after irradiation [57]. Similarly, in the present study, we also focused on the cells that have been induced to produce a stemness-like phenotype after irradiation. Unlike the “rare” number of inherent CSCs, the number of cell populations induced to produce a stemness-like phenotype after radiation was large, and our results also indicated that they did not belong to any one specific cluster but rather to a relatively large group. Moreover, in the proposed pseudotime analysis, we found that the changes in stemness-like responsive cells showed complexity and diversity in the irradiation process, which endowed them with more properties such as malignant proliferation, reprogramming of energy metabolism, and the ability to resist the radiation killing effect. Therefore, we suggest that these radiation-induced stemness-responsive cancer cells might be more important than the inherent CSCs for the development of radioresistance. In other words, our study suggests that radioresistant cells originate from radiation-induced stemness-responsive cancer cells. In addition, radiation-induced stemness was found not to be a dose-dependent or dose-accumulative consequence but could be triggered by relatively low-dose irradiation in the early stage of radiotherapy. Therefore, stemness-responsive cells have better timeliness and effectiveness in the process of radioresistance establishment. The degree of radiation-induced stemness might serve as an indicator that can predict the effect of radiotherapy and radioresistance. In previous reports, researchers have suggested a high degree of heterogeneity and functional changes in CSCs during the course of treatment and the importance of time points for biomarker assessment [58], which is consistent with our findings. Therefore, we believe that the involvement of CSCs in tumor progression and resistance to treatment is not only due to differences in the content of inherent CSCs but also the heterogeneity of properties and functions at different time points. This is one of the directions worth studying in the future.

Although EGFR is considered a classical and important tumor marker, its role in the development of radioresistance is not clear. We found that EGFR was the core stemness-responsive driver gene by pseudotime analysis and gene interaction analysis, suggesting that EGFR can mediate radiation-induced stemness activation in cancer cells. The Hippo pathway can regulate stemness in cancer cells [33,34,35,36,37]. Based on the KEGG enrichment analysis, we found that the Hippo signaling pathway was significantly up-regulated in the A549-6Gy group, where the stemness-like expression was most pronounced, suggesting that the Hippo signaling pathway is directly involved in radiation-induced stemness enhancement. There are few studies on the regulatory relationship between EGFR and the Hippo signaling pathway. Toshinori et al. demonstrated that EGFR can regulate the Hippo pathway by promoting tyrosine phosphorylation of MOB1 [59]. Reddy et al. found that Hippo signaling can be regulated by EGFR-MAPK signaling and is associated with tumorigenesis [60]. Dan et al. demonstrated that the activation state of the endosomal actin regulator WASH is a central node linking the activation of EGFR and Hippo signaling [61]. In the present study, we not only further proved their regulatory relationship but also found that the EGFR-Hippo signaling pathway was involved in the radiation-induced stemness response and the development of radioresistance.

In clinics, EGFR inhibitors (EGFR-TKI) have already been used as a mature targeted medicine, such as Afatinib, Almonertinib, Brigatinib, etc., which are approved for the treatment of NSCLC [62]. Numerous studies have demonstrated the benefits of EGFR-TKI in combination with radiotherapy. For example, primary tumor radiotherapy prolongs the survival of lung adenocarcinoma patients after disease control with EGFR-TKI, including both oligometastatic and multimetastatic patients [63]. EGFR-TKI + thoracic stereotactic body radiation therapy (SBRT) significantly prolongs progression-free survival (PFS) with tolerable toxicity [64]. Of note, patients receiving osimertinib in combination with chest radiotherapy had a particularly high incidence of second-degree or higher severe radiation pneumonitis compared to patients receiving erlotinib or gefitinib, so the choice of combination regimen should be made with caution [65]. In patients with NSCLC with brain metastases, radiotherapy combined with EGFR-TKI improves treatment response and one-year survival compared with radiotherapy alone [66]. In advanced EGFR-mutant NSCLC with brain metastases, using both EGFR-TKI and whole brain radiation therapy (WBRT) resulted in a longer intracranial PFS (iPFS) than EGFR-TKI alone [67]. However, a case report suggests that the combination of WBRT and afatinib may result in serious skin toxicity [68]. Therefore, more multi-institutional, prospective clinical trials are needed in the future to further explore the combined use of EGFR-TKIs and radiotherapy to better guide clinical treatment. Based on the present study, the inhibition of EGFR at an early stage of radiotherapy can inhibit the development of radioresistance in cancer cells. Therefore, combining EGFR-TKI during radiotherapy should focus on the time point of administration. We suggest that combining EGFR-TKI at the early stage of radiotherapy might not only produce a longer-term effect that is beneficial to the enhancement of the radiotherapy outcome but also inhibit tumor recurrence due to radiation-induced stemness-responsive cells. In addition, radiation-induced stemness-responsive cancer cells should be included in the effect evaluation system of EGFR-targeted therapy combined with radiotherapy.

This study also has some limitations. Firstly, we found radiation-induced stemness-responsive cancer cells by single-cell sequencing, but we were unable to isolate them to quantify them or carry out further biological studies. Secondly, we mainly used cell lines for the whole experimental design to focus our sight on cancer cells and to try to find a clear reason for the formation of radioresistance. In addition to the cell line level, we cannot determine which other cells in the tumor microenvironment (TME) are involved in the development of radioresistance and how they work. In recent years, more and more attention has been paid to the study of whether other cells in the TME are involved in the formation of radioresistance. Leyao et al. summarized the progress of the study of CSCs in radioresistance linked to the regulation of autophagy and immune network regulation and illustrated that radiation affects the infiltration of M2-type tumor-associated macrophages (TAMs), promoting EMT and facilitating the activation of PD-1/PD-L1, which reduces the T cell secretion, leading to immune evasion and promoting radioresistance [69]. Similarly to our study, a study on cholangiocarcinoma sampled at time points of tumorigenesis and analyzed by single-cell sequencing showed dynamic interactions of cancer stem-like cells with immune cells and stromal cells at different time points, revealing dynamic changes in the transcriptome during cholangiocarcinoma progression [70]. Therefore, we believe that focusing on the other cells in the TME is also necessary, and our next experimental plan is underway to add more evidence and references to this research.

In summary, our study revealed the dynamic evolution of cell subpopulation in cancer cells during fractionated irradiation and identified a subpopulation of “radiation-induced stemness-responsive cancer cells” in NSCLC cells. During fractionated irradiation, the early stage of irradiation could determine the developmental trend and destiny of the radiation-induced stemness-responsive cancer cells, which will shed new light on the strategy of radiotherapy alone and even radiotherapy combined with other methods. Radiation-induced stemness-responsive cancer cells might provide a prognostic indicator of the efficacy of radiotherapy, a potential target for the development of novel radiotherapy protocols, and, hopefully, a reference for the establishment of integrated protocols for tumor diagnosis and treatment.

## 4. Materials and Methods

### 4.1. Cell Culture

Human NSCLC A549 cell was purchased from Cos9X Bio (Suzhou, China). Cells were cultured in 1640 medium (MeilunBio^®^, Dalian, China) supplemented with 10% fetal bovine serum (FBS) (ExCell Bio, Suzhou, China) in a humidified incubator at 37 °C under 5% CO_2_. Phosphate-buffered saline (PBS) used for cell passaging was purchased from Servicebio (Wuhan, China); EDTA-containing trypsin was purchased from MeilunBio (Dalian, China). All experiments were performed with mycoplasma-free cells. The A549 cell radioresistance modeling was carried out following the steps described in Section 2.1.1 as shown in Figure 1A. This model was repeated after validation of radioresistance and sampling was performed during the process, which was carried out as described in the steps in Section 2.1.1, as shown in Figure 4A. During the modeling process, constant passages were maintained to ensure that each sample originated from the same maternal line.

### 4.2. X-Ray Irradiation

Both cells and nude mice in this study were irradiated using an X-ray generator (X-Rad 225, PXI Inc., Menifee, CA, USA) at dose rate of approximately 1Gy/min at the Institute of Modern Physics, Chinese Academy of Sciences.

### 4.3. Cell Viability Assay

The colony formation assay was performed as previously reported [71]. A549 cells and A549-RR cells were irradiated with 4, 6, and 8 Gy and inoculated with 500 cells in the unirradiated groups and 1000 cells in the irradiated groups, with a minimum of 3 duplicate dishes in each group. After about 2 weeks of incubation, the cells were stained with crystal violet and, finally, the clonal sites were counted. The CCK-8 reagent used in this study was purchased from TargetMol^®^ (Boston, MA, USA). After 4, 6, and 8 Gy radiation of A549 cells and A549-RR cells, 5000 cells were added to each well of a 96-well plate with 50 μL of medium, and 6 replicate wells were added to each group. After incubation for 24, 48, and 72 h, 10 μL of CCK-8 reagent was added. After being protected from light for 1 h, the OD values were measured at 450 nm using an enzyme-labeled instrument (InfiniteM200, Männedorf, Switzerland).

### 4.4. Transwell Assay

The invasive capacity of cells was assayed using Transwell chambers containing Matrigel. A total of 5 × 10^4^ A549 and A549-RR cells were, respectively, resuspended in FBS-free medium and filled into the upper chamber, and the lower chamber was filled with 500 mL of complete medium containing 10% FBS. Twenty-four hours later, the cells were fixed with 4% paraformaldehyde across the stromal gel and stained with 0.5% crystal violet. Three independent experiments were performed in which five random fields of view were photographed and quantified under an inverted microscope.

### 4.5. Cell Transfection

siRNA for EGFR knockdown was designed and synthesized by Genecarer Bio. (Xi’an, China). Transfection was performed in six-well plates at moderate cell confluence (at a 50–60% confluence) by adding the siRNA and transfection reagent (Invitrogen Lipofectamine 3000, Waltham, MA, USA) according to the instructions.

### 4.6. γ-H2AX Foci Test

To detect the extent of DNA damage caused by radiation, the amount of DNA damage marker γ-H2AX (i.e., phosphorylated H2AX) was detected by immunofluorescence staining after 1 h and 6 h of X-ray irradiation for A549-RR and A549 cells, respectively. Specific operations were performed according to the guidelines of DNA Damage Assay Kit by γ-H2AX Immunofluorescence produced by Beyotime Biotechnology (C2035S, Shanghai, China). Confocal images were acquired using a Zeiss LSM-700 confocal microscope (Carl Zeiss, Jena, Germany) equipped with a Plan-Apo 40 £ 1.3 NA oil-immersion objective. Images were edited in Adobe Illustrator 2022 (San Jose, CA, USA).

### 4.7. Western Blot Assay

After lysis of cells with RIPA buffer (Beyotime Biotechnology, P0013C, Shanghai, China), Western blot experiments were performed with the corresponding antibodies as previously described [72].

### 4.8. In Vivo Experiments

Six-week-old female nude mice were purchased from GemPharmatech Co., Ltd. (Nanjing, China). A total of 24 nude mice were randomly divided into two groups and inoculated with A549 and A549-RR cells near the axilla at 5 × 10^6^ cells/each, respectively. Tumor volume was examined and after about two weeks, 2 nude mice in each group with too large or too small tumor volumes were excluded, leading to a total of 20 mice for the next step of the experiment. Before irradiation treatment, each group of nude mice was divided into two groups with equal mean tumor volumes, i.e., the irradiated and non-irradiated groups had equal mean initial tumor volumes. That is, 20 nude mice were divided into 4 groups of 5 mice each. The groups were as follows: A549-0 Gy group, A549-6 Gy group, A549-RR-0 Gy group, and A549-RR-6 Gy group. After irradiation, they were observed for three weeks and then executed, and the tumor tissues were dissected out and fixed in 4% paraformaldehyde for further study. All animal experiments were approved by the Laboratory Animal Welfare Ethics Committee of the Institute of Modern Physics, Chinese Academy of Sciences (Grant No. 2023(002)), and were conducted in accordance with the approved protocols and other relevant guidelines.

### 4.9. Single-Cell Sequencing

Cells were added to the cell suspension at a concentration of 1000 cells/µL to the Genomics Chromium™ (10× Genomics, Pleasanton, CA, USA). Samples were processed as directed and sequenced on the Illumina Nova6000 platform using double-ended 150 bp. Data downloaded from the Illumina platform were used for bioinformatics analysis. All analyses were performed by Applied Protein Technology Co., Ltd. (Shanghai, China).

### 4.10. Seahorse Analysis for Measuring Cellular Energy Metabolism Phenotypes

Cells were inoculated at 12,000 cells/well with complete medium in Seahorse XFp cell culture miniplate (Seahorse Bioscience, Bothell, WA, USA). After overnight incubation, the medium was replaced with Seahorse XF assay medium (Seahorse Bioscience, Bothell, WA, USA) supplemented with 2 mM glutamine, 10 mM glucose, and 1 mM sodium pyruvate. After incubation in a CO_2_-free incubator for 60 min, measurements were made with the Seahorse XFp cell energy phenotype test kit (Seahorse Bioscience, Bothell, WA, USA) and Seahorse XFp analyzer (Seahorse Bioscience, Bothell, WA, USA).

### 4.11. Protein Screening Array

Protein screening array detection was performed by RayBiotech, Inc. (Norcross, GA, USA) using the RayBio^®^ Label-Based (L-Series) Human Hippo Pathway Screening Array (AAH-BLG-HIP-4 (4 Sample Kit)). Biotin-labeled samples were added to glass slides pre-printed with capture antibodies. The slide was incubated to allow binding of target proteins. Then, streptavidin-conjugated fluorescent dye was applied to the array. Finally, the glass slide was dried, and the signals were observed using a laser fluorescence scanner. The raw data obtained from the scan were processed by the Raybiotech software www.raybiotech.com (Norcross, GA, USA) for background removal and normalization and then analyzed for differential proteins and functional enrichment.

### 4.12. Data Analysis

Data were subjected to at least 3 independent experiments. The results of γ-H2AX foci as well as the Western blot experiments were quantified using ImageJ 2 software (NIH, Bethesda, MD, USA). Statistical analysis of the data was performed using GraphPad Prism 9 software (San Diego, CA, USA). Values of *p* < 0.05 were considered statistically significant.

## Data Availability

All data generated or analyzed during this study are included in this article and Supplementary Materials. Additional data are available from the corresponding author upon reasonable request.

## Supplementary Materials

The following supporting information can be downloaded at https://www.mdpi.com/article/10.3390/ijms26041433/s1.

## Abbreviations

The following abbreviations are used in this manuscript:

CSC	Cancer stem cell
ECAR	Extracellular acidification rate
EGFR	Epidermal growth factor receptor
EGFR-TKI	Epidermal growth factor receptor inhibitor
FBS	Fetal bovine serum
GO	Gene Ontology
GSH	Glutathione
KEGG	Kyoto Encyclopedia of Genes and Genomes
MSC	Mesenchymal stem cell
NSCLC	Non-small cell lung cancer
OCR	Oxygen consumption rate
PBS	Phosphate-buffered saline
PFS	Progression-free survival
ROS	Reactive oxygen species
SBRT	Stereotactic body radiation therapy
TAMs	Tumor-associated macrophages
TAZ	Transcriptional coactivator with PDZ-binding motif
TGI	Tumor growth inhibition
TME	Tumor microenvironment
UMAP	Uniform Manifold Approximation and Projection
WBRT	Whole brain radiation therapy
YAP	Yes-Associated Protein
