# Supplementary material for: Single-Cell Sequencing Reveals the Role of Radiation-Induced Stemness-Responsive Cancer Cells in the Development of Radioresistance
Source: Int J Mol Sci. 2025 Feb 8;26(4):1433. doi: 10.3390/ijms26041433 (PMC11855645; doi:10.3390/ijms26041433)
Supplement: Supplementary file 1 [file ijms-26-01433-s001.zip › ijms-3455597-supplementary.pdf]

1. Stem cell-related genes expressed on the UMAP map

   In addition to the distribution of the four genes presented in Figure 5(A), the following are the distributions of the other analyzed stem cell-related genes, which also correspond to the gene in Figure 5(B).

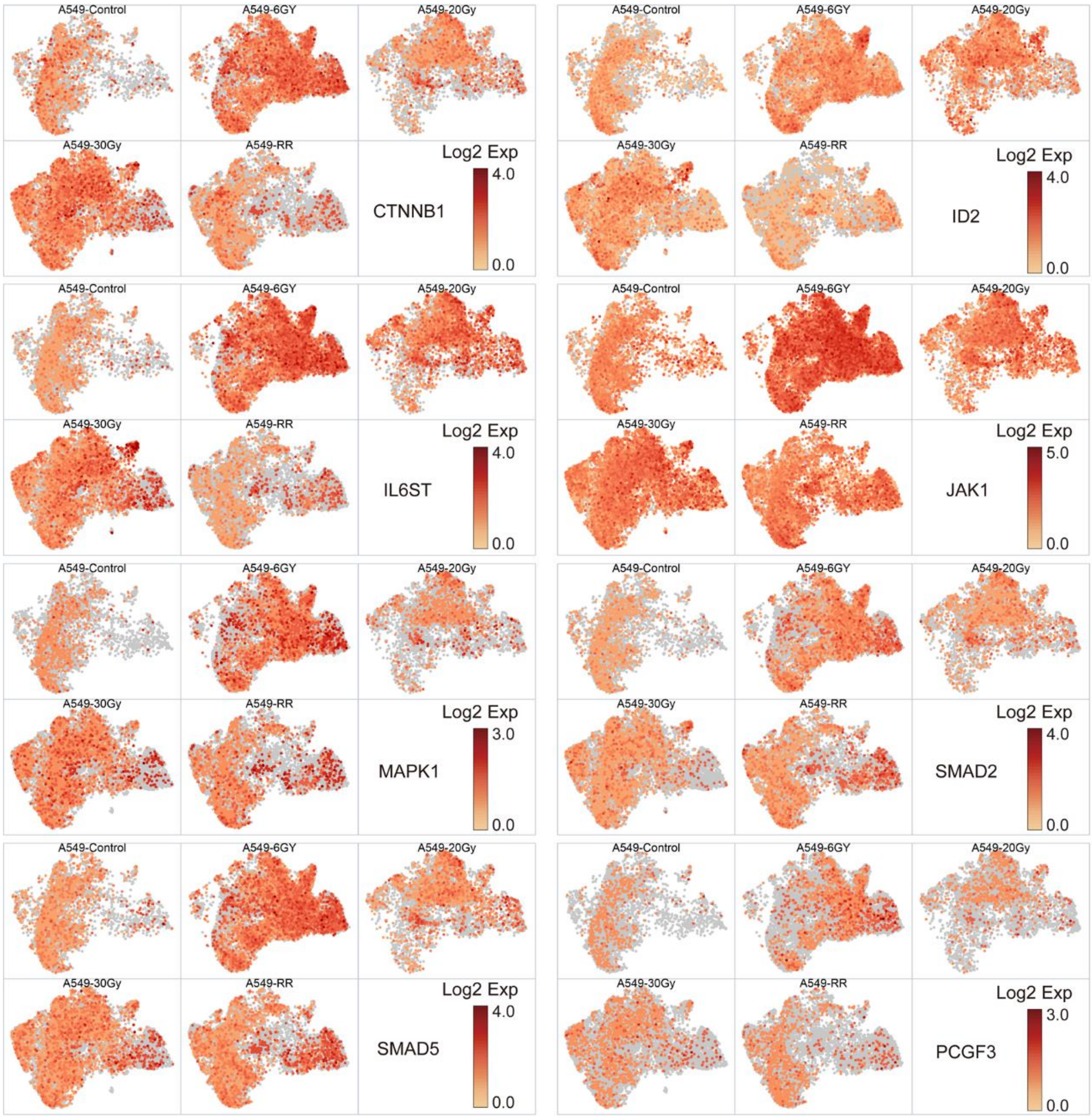

Figure S1. Stem cell-related genes expressed on the UMAP plot.

2. Gene set for scoring stemness

   In the main text we mentioned scoring each group with several genes to measure their stemness-like expression levels, by which we obtained the results in Figure 5(D). The following list shows the names of the genes used for scoring.

Table S1. List of gene set for scoring stemness

Gene name	Gene name
APC	KLF4
BMI1	MAPK1
BMP4	MYC
BMPR1A	PCGF2
CTNNB1	PCGF3
ID2	PCGF5
IL6ST	PCGF6
JAK1	SMAD2
JAK2	SMAD5
JARID2	WNT3

3. Gene set for scoring Hippo signaling pathway

As mentioned in the main text, we used a gene set to score each group for the Hippo signaling pathway, and the results obtained are shown in Figure 8(E). The names of the genes used to score the Hippo signaling pathway are listed below.

Table S2. List of gene set for scoring Hippo signaling pathway

Gene name	Gene name	Gene name	Gene name
ACTG1	CTNNA3	MPP5	WWTR1
FGF1	CTNNB1	NF2	TCF7
AJUBA	CCND1	PAK1	TCF7L1
ALK6	DLG4	PARD3	TGFB1
HUMAFP	DVL1	PARD6A	TGFB2
AMH	DVL2	PARD6B	TGFB3
AREG	DVL3	PPP1CA	TGFBR1
APC	CDH1	PPP1CC	TGFBR2
AXIN1	FBXW11	PPP2CA	TP73
AXIN2	FZD1	PPP2CB	WNT1
BMP2	FZD2	PPP2R1A	WNT10A
BMP4	FZD3	PPP2R1B	WNT10B
BMP5	FZD4	PPP2R2A	WNT11
BMP6	FZD5	PPP2R2B	WNT16
BMP7	FZD6	PPP2R2C	WNT2
BMP8	FZD7	PRKCI	WNT2B
BMP8B	FZD8	PRKCZ	WNT3A
BMPR1A	GDF5	RASSF1	WNT4

BMPR2	GDF7	RASSF2	WNT5A
BTRC	MSTN	SERPINE1	WNT5B
CCN2	GLI2	SMAD1	WNT6
CCND2	GSK3B	SMAD2	WNT7A
CCND3	ID1	SMAD3	WNT7B
BIRC2	ID2	SMAD4	WNT8B
BIRC3	ITGB2	SMAD7	WNT9A
CMYC	LEF1	SOX2	WNT9B
CSNK1D	LIMD1	STK3	WWC1
CSNK1E	MOB1B	BIRC5	YAP1

4. Map of the antibodies of the antibody array

As mentioned in the main text, we used a Hippo pathway antibody array to measure the expression of different groups of Hippo pathway-related proteins and demonstrated the original fluorescence response on the antibody array as shown in Figure 9(B). The following table lists the names of the antibodies corresponding to this array.

Table S3. Map of antibody arrays -- AAH-BLG-HIP

9	8	7	6	5	4	3	2	1	
YWHAQ	WNT5A	TGFB1	PPP2R2C	MPP5	FZD5	CDH1	BMP2	POS1	1
YWHAQ	WNT5A	TGFB1	PPP2R2C	MPP5	FZD5	CDH1	BMP2	POS1	2
YWHAZ	WNT5B	TGFB2	PRKCI	MYC	FZD6	CSNK1D	BMP4	POS2	3
YWHAZ	WNT5B	TGFB2	PRKCI	MYC	FZD6	CSNK1D	BMP4	POS2	4
	WNT6	TGFB3	PRKCZ	NF2	FZD7	CSNK1E	BMP5	POS3	5
	WNT6	TGFB3	PRKCZ	NF2	FZD7	CSNK1E	BMP5	POS3	6
	WNT7A	TGFBR1	RASSF1	PAK1	FZD8	CTNNA3	BMP6	Neg	7
	WNT7A	TGFBR1	RASSF1	PAK1	FZD8	CTNNA3	BMP6	Neg	8
	WNT7B	TGFBR2	RASSF2	PARD3	GDF5	CTNNB1	BMP7	ACTG1	9
	WNT7B	TGFBR2	RASSF2	PARD3	GDF5	CTNNB1	BMP7	ACTG1	10
	WNT8B	TP73	SERPINE1	PARD6A	GDF6	DLG4	BMP8A	AFP	11
	WNT8B	TP73	SERPINE1	PARD6A	GDF6	DLG4	BMP8A	AFP	12
	WNT9A	WNT1	SMAD1	PARD6B	GDF7	DVL1	BMP8B	AJUBA	13
	WNT9A	WNT1	SMAD1	PARD6B	GDF7	DVL1	BMP8B	AJUBA	14
	WNT9B	WNT10A	SMAD2	PPP1CA	GLI2	DVL2	BMPR1A	AMH	15
	WNT9B	WNT10A	SMAD2	PPP1CA	GLI2	DVL2	BMPR1A	AMH	16
	WWC1	WNT10B	SMAD3	PPP1CC	GSK3B	DVL3	BMPR1B	APC	17
	WWC1	WNT10B	SMAD3	PPP1CC	GSK3B	DVL3	BMPR1B	APC	18
	WWTR1	WNT11	SMAD4	PPP2CA	ID1	FBXW11	BMPR2	AREG	19
	WWTR1	WNT11	SMAD4	PPP2CA	ID1	FBXW11	BMPR2	AREG	20
	YAP1	WNT16	SMAD7	PPP2CB	ID2	FGF1	BTRC	AXIN1	21
	YAP1	WNT16	SMAD7	PPP2CB	ID2	FGF1	BTRC	AXIN1	22
	YWHAB	WNT2	SOX2	PPP2R1A	ITGB2	FZD1	CCN2	AXIN2	23
	YWHAB	WNT2	SOX2	PPP2R1A	ITGB2	FZD1	CCN2	AXIN2	24
	YWHAE	WNT2B	STK3	PPP2R1B	LEF1	FZD2	CCND1	BIRC2	25
	YWHAE	WNT2B	STK3	PPP2R1B	LEF1	FZD2	CCND1	BIRC2	26
	YWHAG	WNT3A	TCF7	PPP2R2A	LIMD1	FZD3	CCND2	BIRC3	27
	YWHAG	WNT3A	TCF7	PPP2R2A	LIMD1	FZD3	CCND2	BIRC3	28
	YWHAH	WNT4	TCF7L1	PPP2R2B	MOB1B	FZD4	CCND3	BIRC5	29
	YWHAH	WNT4	TCF7L1	PPP2R2B	MOB1B	FZD4	CCND3	BIRC5	30

(This table has been rotated 90° due to vertical length constraints. The orientations in figure 5 (D) are: left-right corresponds to the right-hand side numbered 1-30 in this table; top-bottom corresponds to the top numbered 1-9 in this table.)

5. Differential proteins in the top100 with gene names compared between the two groups.

   Since the text of the gene names in the original heatmap of differential proteins was too small, we made them into pie charts for aesthetic purposes and displayed them in

Figure 9(C) in the main text. The original top100 heatmap of differential genes with gene names is shown below. Up-regulated proteins are shown in red and down-regulated proteins in blue.

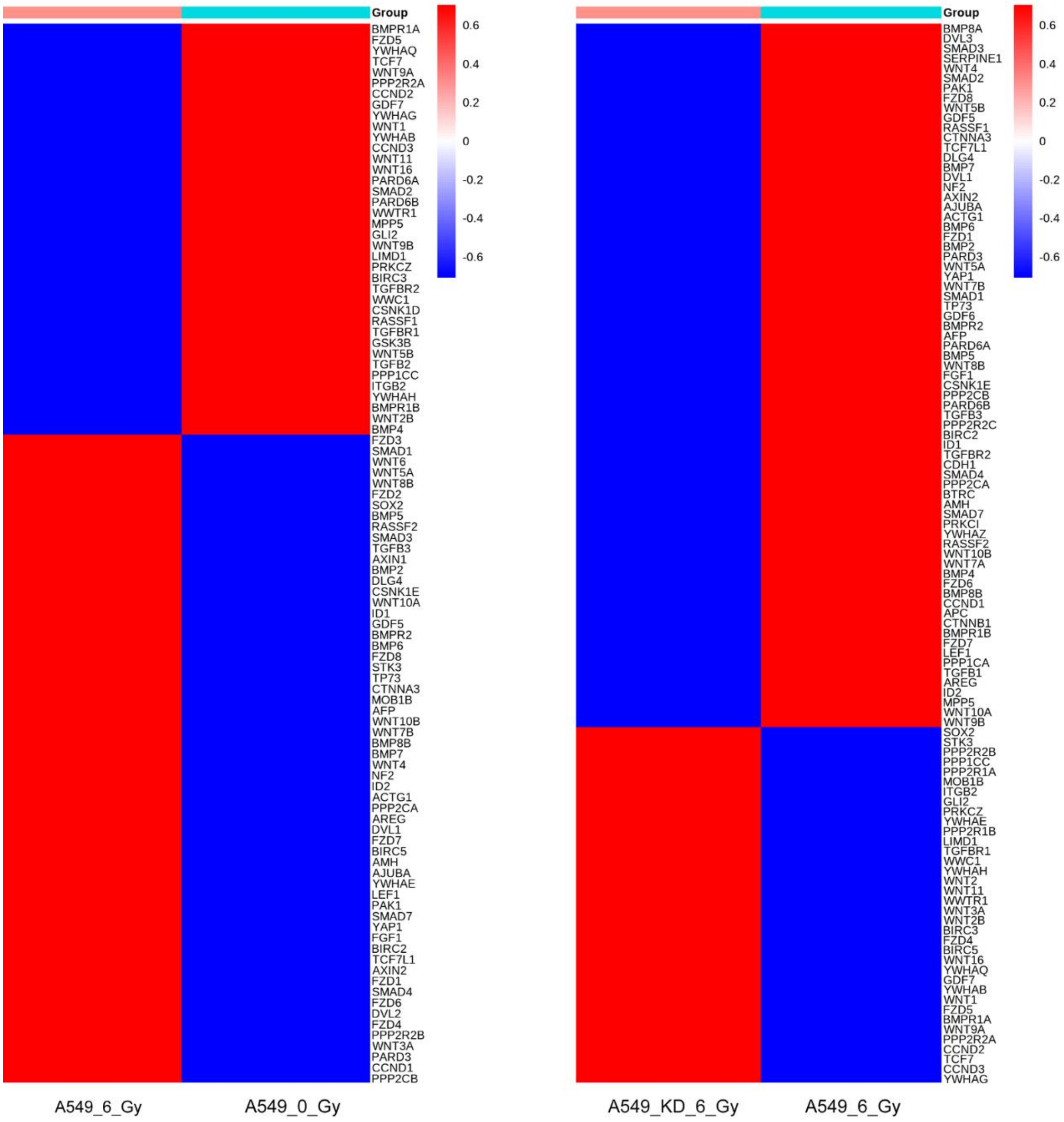

Figure S2. The top100 heatmap of differential genes with gene names between two groups.
